# An Optimization Method for Lightweight Rock Classification Models: Transferred Rich Fine-Grained Knowledge

**DOI:** 10.3390/s24134127

**Published:** 2024-06-25

**Authors:** Mingshuo Ma, Zhiming Gui, Zhenji Gao, Bin Wang

**Affiliations:** 1 Faculty of Information Technology, Beijing University of Technology, Beijing 100124, China; mamingshuo@emails.bjut.edu.cn (M.M.); zmgui@bjut.edu.cn (Z.G.); 2 Integrated Natural Resources Survey Center, CGS, No. 55 Yard, Honglian South Road, Xicheng District, Beijing 100055, China; 3 Technology Innovation Center of Geological Information Engineering of Ministry of Natural Resources, Beijing 100055, China

**Keywords:** rock images classification, contrastive learning, knowledge distillation

## Abstract

Rock image classification represents a challenging fine-grained image classification task characterized by subtle differences among closely related rock categories. Current contrastive learning methods prevalently utilized in fine-grained image classification restrict the model’s capacity to discern critical features contrastively from image pairs, and are typically too large for deployment on mobile devices used for in situ rock identification. In this work, we introduce an innovative and compact model generation framework anchored by the design of a Feature Positioning Comparison Network (FPCN). The FPCN facilitates interaction between feature vectors from localized regions within image pairs, capturing both shared and distinctive features. Further, it accommodates the variable scales of objects depicted in images, which correspond to differing quantities of inherent object information, directing the network’s attention to additional contextual details based on object size variability. Leveraging knowledge distillation, the architecture is streamlined, with a focus on nuanced information at activation boundaries to master the precise fine-grained decision boundaries, thereby enhancing the small model’s accuracy. Empirical evidence demonstrates that our proposed method based on FPCN improves the classification accuracy mobile lightweight models by nearly 2% while maintaining the same time and space consumption.

## 1. Introduction

The classification of rock types is an essential aspect of geological studies, and the precise and efficient categorization of rock images is of considerable importance. This classification is regarded as a task of fine-grained visual categorization [1].

Deep learning-based computer vision technologies augment image sensors by processing and interpreting visual data, enabling machines to respond to their environment in a manner akin to human vision. Advanced algorithms [2] extract valuable rock feature information from sensor data, then enhance image quality to aid in image recognition and classification, effectively extending the utility of image sensors beyond simple image capture.

Despite marked advancements in general image classification through convolutional neural networks (CNNs) in recent years [3], the fine-grained classification of rock images remains a significant challenge owing to the pronounced similarities among subclasses.

Recent advances have been made in the automated classification of rock images. Chatterjee et al. [4] extracted textural features from rock images and applied a Support Vector Machine (SVM)-based approach for regression prediction utilizing these features. Liang et al. [2] employed CNN-based methods, while Guojian [5] introduced a rock slice image classification technique using residual networks. Pascual et al. [6] utilized a three-layer CNN architecture to classify rock images, resulting in enhanced recognition precision. To bolster the learning of discriminative features and mitigate overfitting, data augmentation strategies have been integrated into fine-grained image classification frameworks. Zhao et al. [7] advocated for the enrichment and augmentation of rock image data using Generative Adversarial Networks (GAN), aiming to enhance model performance by obtaining superior data samples.

### 1.1. Common Fine-Grained Image Classification Techniques

Due to the impact of the camera’s location and angle and the resolution of the camera equipment, the objects to be classified in rock images typically have arbitrary locations and do not fill the entire image. The localization of classification targets can eliminate the interference of other elements in the background of the image. Most localization methods use bounding boxes to present the position of objects and mark the regions in the images that contain objects. Several localization methods determine the position of objects based on features generated by convolutional neural networks [8,9,10,11]. R-CNN approaches [10] involve the use of techniques such as selective search [12] to generate several candidate regions in the detection image and extract the corresponding feature vector from each region, with the most accurately classified region then identified as the target region. This approach requires manual annotation of the bounding boxes of the object parts, which consumes a significant amount of time and computational resources. Therefore, methods that use weakly supervised localization [13,14,15,16] have been introduced, such as RA-CNN [16], which continuously enlarges the area of interest in the convolutional layer and uses the enlarged image as input; the obtained classification result is then considered a part of the final result. S3N [17] determines the object region based on the maximal response location for each category in the image. While these methods achieve satisfactory results without the need for bounding box annotations, they require a two-stage process of localization and classification, and the presence of multiple inputs and networks can lead to complex models with lower efficiency.

In rock image samples, the classification of rock types largely depends on complex visual characteristics such as color and texture variations. High-quality features are particularly important in fine-grained image classification, as the visual differences between fine-grained images are often minimal. Building a robust feature representation has been extensively studied for fine-grained image classification. One of the earliest representative methods is Bilinear CNN [18], which aggregates features extracted by two CNNs to generate higher-order features representing more complex and higher-dimensional characteristics. Subsequent methods inspired by this approach include several methods [19,20,21,22,23] that rely on multiple feature extraction modules for multi-view image samples that are cropped, erased, masked, and zoomed for each module’s input. These methods can extract richer feature representations and achieve better results, but cannot ascertain which features truly differentiate between classes or which are unique within a class. Rao [24] proposed a causal inference-based attention learning method that encourages the network to learn more effective visual attention. Inspired by this, fine-grained image classification methods based on contrastive learning have been proposed. Contrastive learning can obtain key features by comparing pair of images in order to distinguish them; this approach is especially effective for fine-grained classification, as it is able to detect subtle differences. CIN [20] uses the channel correlation between samples to bring positive pairs closer through channel interaction while pushing negative pairs apart, whereas PCA-Net [21] and API-Net [22] learn common and distinguishing features through channel interactions by simultaneously inputting positive/negative sample pairs. Fine-grained image classification methods based on contrastive learning have achieved the best results to date; however, the existing problem is that while the similar features of samples within the same class and different features of samples from different classes are used as bases for discrimination, these are not correct reasons for differentiation. For example, in a case where a pair of images belonging to different classes share the same background, the model might improperly use this similarity as a basis for classification.

### 1.2. Lightweight CNNs Models for In Situ Rock Classification

Although this kind of method solves the problem of professionals with rich geological knowledge and experience being in very high demand, the process of rock identification and classification must still take place within the laboratory [25]. Moreover, the research results cannot be directly applied by geological surveyors working in the field [26], preventing effective real-time feedback. Wang et al. [27] designed a lightweight deep neural network with depthwise separable convolutions based on MobileNets and the transfer learning method, which they used to develop a lithology recognition model for rock images in the field. However, their model was not optimized or compressed in detail, and they did not compare it to other CNN-based models. The CNN backbone often refers to feature extraction layers and the generation of a representation vector for use in downstream tasks such as classification. Baraboshkin et al. [28] compared the effectiveness and efficiency of four types of mainstream CNN backbone models and discussed the importance of convenience and productivity in rock classification. However, their proposed method cannot be deployed on mobile devices. Fan et al. [29] proposed a rock classification model based on MobileNet and SqueezeNet architectures. They tested the model on mobile devices and presented detailed time and space consumption, proving that their model is applicable in the field. The above methods attempt to improve classification accuracy and efficiency by employing different CNN backbones, but do not consider two points: first, in fine-grained classification tasks it is important to preserve the rich features in rock images while applying a lightweight model, as multiple features can determine correctness; second, additional model optimizations such as compression and distillation need to be carried out in order to improve their potential and efficiency.

### 1.3. Improving Lightweight Deep Neural Networks by Knowledge Distillation

Although some fine-grained image classification algorithms have addressed the limitation of requiring additional annotated information and have achieved target extraction under unsupervised or weakly supervised conditions, balancing model accuracy and model size remains challenging. Pursuing high accuracy necessitates increased complexity and computational demands, leading to longer inference and prediction time costs. In the context of rock image classification, which often involves mobile environments, it is essential to address both size and accuracy. One of the current approaches for achieving this goal involves improving the classification accuracy of small models without expanding their size.

### 1.4. Refining the Decision Boundaries of Neural Networks

Using the trained model to predict all coordinate points in the sample space, the boundaries between different classes can be observed across all points in the sample space, ultimately yielding the model’s decision boundaries. Activation boundaries refer different states of neurons in one layer between the deep network and formed regions. The shape of the decision boundary and the distance of sample points from the boundary are crucial in fine-grained classification tasks. Existing large-margin learning methods [30] mostly focus on fine-grained classification problems, such as face recognition and voiceprint recognition. The goal of large-margin learning algorithms is to increase the distance between sample points and the decision boundary. The advantages of the resulting large-margin classifiers include better generalization and robustness to input perturbations, which are highly effective for situations involving a large number of fairly similar samples and a few difficult samples, as is the case in fine-grained classification tasks. In many classical machine learning algorithms, such as Support Vector Machine, the distribution of sample points in the sample space is fixed and it is only necessary to solve for the optimal decision boundary. However, in deep learning models both the representation of samples and the decision boundaries are learned through the backpropagation algorithm. For nonlinear deep networks, the distances between the sample points and the decision boundary are often replaced by approximate values, making optimization of the decision boundary in deep learning more complex and difficult to compute.

There are several methods for expanding the margin in the current deep learning literature. Sokolic et al. [31] aimed to limit the local variations of the model by minimizing the Lipschitz constant such that it does not exceed a certain constant by using L2 regularization during the model optimization process to prevent arbitrary expansion of model parameters, thereby enhancing the model’s generalizability. Additionally, introducing a margin in the model’s decision space (such as the probability space of model outputs) or representation space can be a more direct approach. S. Sun et al. [32] noted that using softmax combined with cross-entropy as the loss function does not inherently maximize the margin, and advocated for an additional penalty term to encourage a larger margin. In the field of face recognition, large margin losses based on softmax have been widely discussed, including in the context of L-Softmax [33] and a unified framework for softmax-based margin loss [34]. Furthermore, increasing the margin in the representation space is a common approach, with local linearization [35] as one representative method. By analyzing scenarios where sample perturbations lead to incorrect predictions, then combining the results with Taylor expansion, it is possible to approximate the specific form of the perturbations, which can serve as a representation of the margin.

Similar to large-margin learning, metric learning concentrates on learning a distance function in order to effectively measure similarities and differences between samples. Metric learning aims to learn a distance function tailored to the specific task. This distance function is designed such that similar samples are closer to each other and dissimilar samples are farther apart. In deep neural networks, metric learning measures the similarity of pairs of samples through a loss function, resulting in better learning of classification decision boundaries and improved model generalizability. Wang et al. [36] utilized a sum-based margin in the cosine space, setting the decision boundary within the cosine space so as to avoid the issue of inter-class samples being unconstrained by the angular distance. The center loss [37] adds a regularization term on top of the softmax loss, pushing apart different class samples in the feature space.

In neural networks, the activation state of the neurons is very important. Montufar [38] proposed that neural networks represent complex functions with combinations of activation boundaries. Pan and Srikumar [39] demonstrated that the decision boundaries of neural networks are composed of combinations of these activation boundaries. Therefore, in knowledge distillation, transferring activation boundary information from teachers to students can convey more crucial classification information to the student models, especially in fine-grained classification problems. This is because models for fine-grained classification often have more complex decision boundaries, which is one of the bases for the methods adopted in this paper.

**Figure 1:**
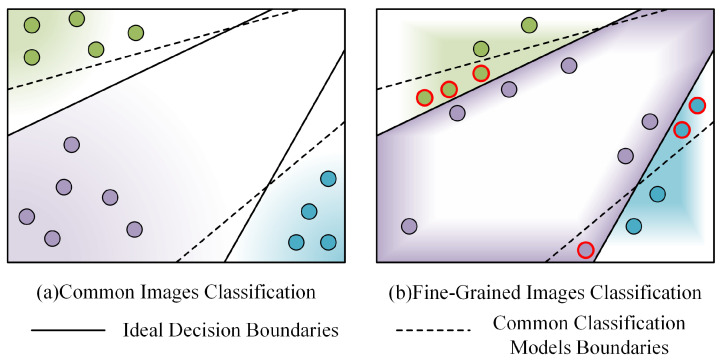
Instances that are easily confused in fine-grained image classification.

Figure 1 illustrates the misclassifications that arise from decision boundaries in general and fine-grained image classifications; the dots represent samples, the solid lines represent the ideal decision boundary of a small model, and the dashed lines represent the model’s actual decision boundary. The different shades of background color indicate the model’s activation response to a certain class; in (a), this refers to general image classification, while in (b) it refers to fine-grained image classification, with misclassified samples marked by red circles.

The key challenges in fine-grained image classification include: (1) considerable intra-class variance, as images of identical rock types may vary in background, perspective, and scale, leading to significant feature discrepancies within the same class; (2) minimal inter-class variance, characterized by highly similar samples among rock subclasses that share hues, textures, and shapes and are only distinguishable by nuanced differences; and (3) constrained recognition conditions in situ, as in field exploration, where limitations in image clarity and mobile device capabilities impede accuracy. Notably, small models on mobile devices often fail to align with the decision boundaries of their larger counterparts, significantly diminishing the resulting fine-grained classification accuracy. Accurately modeling the decision boundaries of larger high-accuracy models remains a formidable challenge. Table 1 shows the advantages and disadvantages of different rock image classification approaches.

This paper presents the design of a Feature Positioning Contrast Network (FPCN) model to effectively mine the critical features that dictate rock types. This approach distinguishes the essential features among rock classes and identifies unique characteristics of specific rock types. The model leverages these features to refine decision boundaries during the knowledge distillation process forin order to develop compact classification models, thereby boosting their performance. The proposed approach involves the following tasks: (a) Without relying on supplementary annotations for rock image location frames, the algorithm captures the fundamental position contours of the main rock body through feature maps produced by a pretrained model. (b) A self-supervised learning strategy is employed that hinges on contrastive learning within and between rock image classes in order to discern the pivotal features of rock types while concurrently filtering out non-essential comparative data. (c) By applying knowledge distillation within the FPCN model, the decision boundaries of the compact models are enhanced, resulting in improved classification accuracy. Furthermore, the paper includes tests and evaluations of compact rock classification models across various scales.

## 2. Knowledge Distillation-Based Lightweight Model Optimizing Framework

**Figure 2:**
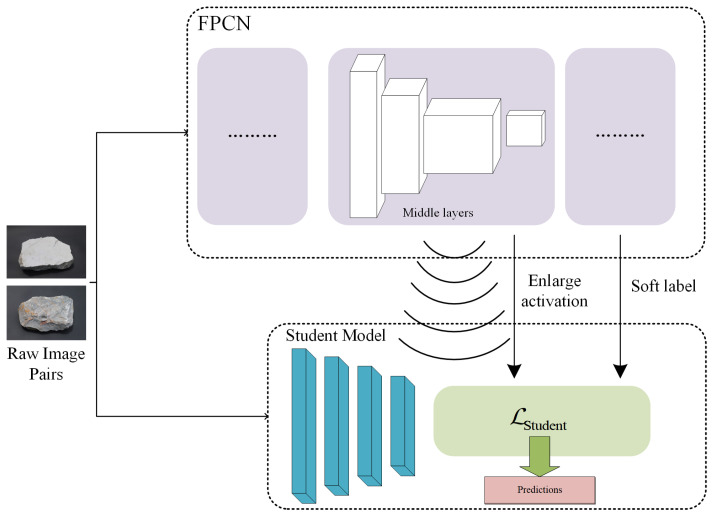
Framework for optimizing lightweight model method based on FPCN.

This chapter introduces the main framework for optimizing lightweight model methods, details the component modules of the FPCN, which contains rich fine-grained features as a teacher model, and describes the feature alignment and decision boundary transfer processes involved in knowledge distillation. As shown in Figure 2, the proposed method primarily involves transferring the activation boundaries of the intermediate convolutional layers of the trained FPCN to the corresponding intermediate layers of the lightweight model through an amplification module. Cross-model correspondence between the shallow and deep layers is achieved through feature alignment and middle transform layers. The final training objectives of the lightweight model include similarity of the activation boundaries, predictive distribution of the FPCN, and the true labels.

### 2.1. Feature Positioning Contrast Network

**Figure 3:**
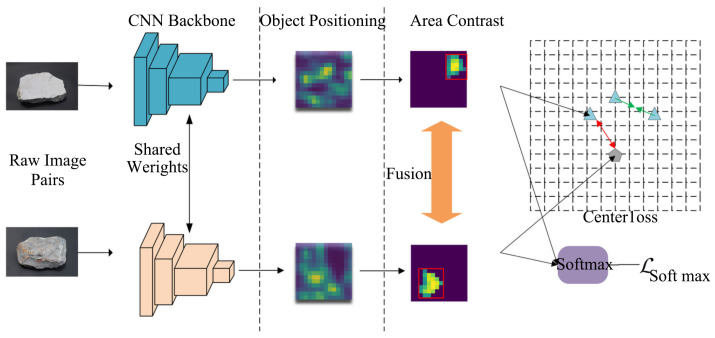
Feature Positioning Contrast Network (FPCN).

As depicted in Figure 3, the FPCN model consists of three main parts: Main Body Positioning, Part Contrast Learning, and Optimization Loss. The goal of the Main Body Positioning module is to identify the approximate area of the object within the image. Given the presence of interference from the background and other objects, which produce irrelevant contrast information, the positioning area aids the contrast module in focusing on the critical contrast locations. Our proposed method for detecting the maximal feature map area does not require additional bounding box annotations, instead obtaining the target position by generating highlighted connected regions in the feature map through a pretrained image detection network.

#### 2.1.1. Feature Maps Area Positioning

**Figure 4:**
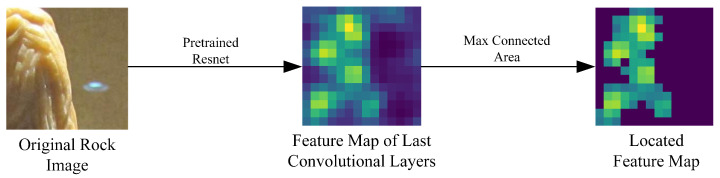
Steps in object positioning.

Given an input image I, $F\in R^{H\times W\times C}$ denotes the feature map obtained after the last convolutional layer of the backbone and H,W,C denote the height, width, and number of channels of the feature map, respectively. Due to interference caused by invalid contrast information from the background and other objects, the localized region can help to focus on the key contrast locations in the contrast module. Our proposed method for detecting the maximum region of the feature map does not require any additional labeling of the position frame; the target location is obtained by generating the highlighted connected regions of the feature map through a pretrained image detection network. The main steps are shown in Figure 4. To account for rock objects with different scales, the size of the maximum connected area is unique and maintains most of the area of the rock, helping to retain the rock features while reducing unrelated background features.

During experiments by Wei et al. [40], it was found that in an image of a single object the main region always corresponds to the position of the largest activation connected area in the feature map of the last convolutional layer. Using the VGG16 [41] model pretrained on the ImageNet dataset, the last layer’s feature map greatly contributes to localization; however, using a single layer for localization in the ResNet model yields poor results with high-resolution image inputs. Due to the varying proportions of the main subject in different images, a single size of feature map cannot represent objects of various scales. Inspired by the Feature Pyramid Networks (FPN) proposed by Lin et al. [42], which have less semantic information in lower layers while providing accurate target locations and richer semantic information but coarser target locations in higher layers, in this paper we use two layers of feature maps to determine the main subject’s position, as shown in Algorithm 1.
**Algorithm 1**: Generation of the largest connected region in feature maps
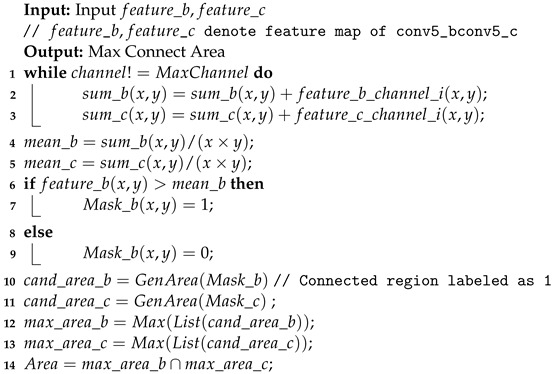



#### 2.1.2. Part Comparison Learning

**Figure 5:**
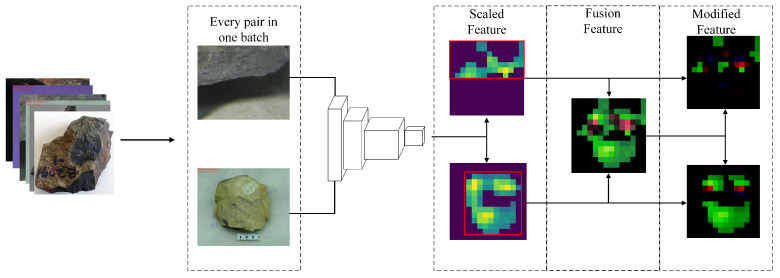
Process of part comparison learning.

In order for the model to learn common features within classes and differential features between classes, we propose a between-objects comparison learning module, as shown in Figure 5. In one training batch, there exist different image pairs of different classes or same class; for every pair in one batch, we first obtain the located feature map through object positioning and input it to f(x), which presents a single linear layer, and output the fusion feature, which contains the similar and unique features for each image (marked in red and green, respectively). The fusion features point out the key feature for judging the rock types. Therefore, the element-wise multiplication between fusion features and scaled features generates modified feature that can activate both the unique and common features of the key features corresponding to the sample.

This is shown in (1), where $z_{i}$ is the feature representation of the *i*th sample, $z_{i+}$ is its positive sample, $z_{i-}$ is its negative sample, · is used to calculate the similarity between the samples, and τ is a temperature parameter that controls the shape of the loss function.

(1) $$L_{c}=-\log\frac{\exp\left(z_{i}\cdot z_{i+}/\tau \right)}{\sum_{i=0}^{K}\exp\left(z_{i}\cdot z_{i-}/\tau \right)}\;$$

The overall optimization process of the model during the training phase is shown in Equation (2), with $L_{class}$ using the cross-entropy loss function between category predictions. The total loss is expressed as the sum of the classification loss and center loss, with λ being a hyperparameter that balances the two losses.

(2) $$L=L_{class}+\lambda L_{c}\;$$

Due to the limitation of batch size in the training process, for a certain sample in each batch the features are compared with other samples in the batch, then the model is trained by calculating the similarity with positive and negative examples. The process is shown in Algorithm 2.
**Algorithm 2**: Feature contrast learning
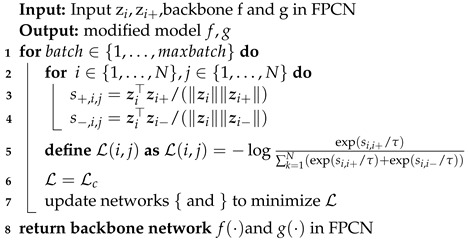



### 2.2. Feature Alignment

Despite the height and width being consistent, it is necessary to add an intermediate layer transformation during the alignment process due to the inconsistent number of channels in the feature maps output by corresponding convolutional layers of different models. In this paper, the intermediate layer transformation refers to the connector structure used in Fitnets proposed by Romero et al. [43]. Assuming the same input image *I*, the feature map sizes obtained from the *i*th convolutional layer which need to be aligned in the teacher model and student model are $H\times W\times C_{a}$ and $H\times W\times C_{b}$, respectively. In order to change only the number of channels, a 1×1 convolution kernel is used in the corresponding convolutional layer to map the channel number $C_{s}$ of the student model to the channel number $C_{t}$ of the teacher model. The convolutional layer used in the *i*th layer is shown in Table 2, where $C_{is}$ and $C_{it}$ represent the number of channels of the feature maps output at the *i*th layer from the student and teacher models, respectively.

In the experiments described in this chapter, the teacher and student models used the activation feature maps of four convolutional layers; thus, the total activation boundary amplification loss function should include the sum of the loss values of each layer. Because the width and height of the feature maps are halved after each block, leading to a reduction in the number of pixels calculated to a quarter of the previous layer, using the L2 norm results in the ratio of each layer’s loss value being reduced to half that of the previous layer. Thus, to balance the feature weights of the four convolutional layers, the loss values of the lower layers are correspondingly reduced, as shown in Equation (3).

(3) $$L_{1}\left(I\right)=\left(L_{C1}/8\right)+\left(L_{C2}/4\right)+\left(L_{C3}/2\right)+L_{C4}$$

### 2.3. Activation Boundary Amplification Module

Due to the importance of activation boundaries for fine-grained tasks, it is insufficient to simply use the neuron’s activation values for distillation constraints. Instead, the neuron’s activation regions should be used. To ensure that the lightweight model’s decision boundaries more closely align with those of the location-contrast network, it is crucial to focus on whether the corresponding neuronal states are the same. The value of if(x) indicates whether a neuron is activated, as shown in Equation (4).

(4) $$if\left(x\right)=\left\{\begin{array}{c} 1,\mathrm{if}\;x=0,\\ 0,\;\mathrm{otherwise} \end{array}\right.$$

To make the decision boundaries of the FPCN and the small model more similar, Equation (5) describes the loss of the small model. The activation state of the neuron in the teacher model if(T(I)) is equivalent to the category label. If the teacher’s neuron is activated, then the response of the student’s neuron should be greater than 0; conversely, if the teacher’s neuron is not activated, then the response of the student’s neuron should be less than 0, as follows:

(5) $$\begin{array}{cc} \; & L(I)=∥\mathrm{if}\;(T(I))⊙\sigma (\mu 1-S(I))+\\ \; & {\left(1-if\left(T\left(I\right)\right)\right)⊙\sigma \left(\mu 1+S\left(I\right)\right)∥}_{2}^{2} \end{array}$$

where ⊙ is the element-wise multiplication between vectors, while 1 represents a vector of length equal to the output size of T(I) with all values being 1. The alternative loss assigns a squared penalty to neurons in the student model that have different activation states from the teacher, without concern for neurons with the same activation values. In our experiments conducted with this method, the layers from which activation values were extracted were all convolutional layers, and each layer’s activation values corresponded to the values of the convolutional layer’s feature maps. Thus, the above formula can be written as shown in Equation (6).

(6) $$L_{1}\left(I\right)=\sum_{i}^{H}\sum_{j}^{W}L(I)$$

Because the training set does not include all data of rock images and new rock images are constantly being produced during exploration, the training goal becomes modeling the relationship between input and output on the existing training dataset. As the training dataset is a sample of the real data distribution, the optimal solution on the training dataset often deviates from the real optimal solution, although it should be noted that this discussion does not consider model capacity. In knowledge distillation, as we already have a model with good performance and strong generalization ability in the FPCN, we can allow the student model to learn the generalization ability of the FPCN directly when using the FPCN to distill the student model. An efficient method for transferring generalization ability is to use the probabilities of categories output by the softmax layer as soft targets.

In the output of the softmax layer, both the positive and negative examples contain a great deal of information; for instance, the probability associated with certain negative labels is much higher than that of others. In the traditional hard-target training process, all negative labels are treated uniformly, meaning that the training method with knowledge distillation provides more information per sample to the student model than the traditional training method.

Finally, the similarity of the activation boundaries, the soft labels for the classification of the teacher model, and the hard labels for the classification of the student model are combined as the comprehensive optimization objective, as shown in Equation (7).

(7) $$L=L_{1}+L_{soft}+L_{hard}$$

## 3. Experiments and Discussion

This chapter first explains the training environment used for training the FPCN and optimizing the lightweight models using the FGVC-ROCK dataset. Then, it compares the FPCN with existing fine-grained image classification models, lists the performance improvements of lightweight models, and discusses how each module contributes to the overall effectiveness.

**FGVC-ROCK Dataset:** The large-scale Fine-Grained Rock Image Dataset contains a variety of rock images with complex backgrounds along with multiple shapes and proportions (Table 3).

**Training FPCN Experimental Setup:** We used the PyTorch deep learning framework and performed training on a computer equipped with an RTX A5000 (24 GB) GPU and 42 GB of memory. FPCN uses a ResNet-101 pretrained on the FGVC-ROCK dataset as its backbone network. First, each input image was resized to 448×448. The training process employed a stochastic gradient descent optimization algorithm (SGD) with a momentum of 0.9, and the training was set for 100 epochs (actually converging around 80–90 epochs) with a batch size of 42 (14 images per class) and an initial learning rate of 0.01. When constructing pairs of input images, mask feature maps and contrast vectors were generated for any two samples in each batch, and the model was optimized as a whole.

**Distillation Experimental Setup:** The experimental environment in this chapter was the same as when training FPCN. The teacher model used the FPCN network trained on the FGVC-ROCK dataset, while the student models included models suitable for deployment on mobile devices, including ResNet18, ResNet34, MobileNet V1, MobileNet V2, ShuffleNet V1, and ShuffleNet V2. The backbone networks of the student models were also pretrained on the ImageNet dataset. The entire distillation model was trained for 100 epochs, with the first 60 epochs used for distillation training, setting an initial learning rate of 0.01, which was reduced to one-tenth every 20 epochs. The setup for the Image Transform is shown in Table 4.

**Convolutional Layer in Different CNN Backbones:** To uniformly denote the corresponding convolutional layers of different models, the labels $C_{1},C_{2},C_{3},C_{4}$ are used to represent the four aligned convolutional layers. The input image *I* has a resolution of 448×448×3. After passing through the first two convolutional layer blocks of the backbone network, a feature map of $\frac{1}{4}$ resolution, denoted as $conv_{2}\left(112\times 112\right)$, is obtained. Subsequently, by passing through the remaining convolutional layers, feature maps of different scales are obtained, namely, $conv_{3}\left(56\times 56\right),\;conv_{4}\left(28\times 28\right)$, and $conv_{5}\left(14\times 14\right)$. The sizes of these feature maps correspond to different convolutional layers in the teacher and student models. By using the similarity of the corresponding feature maps, the student model learns the activation boundaries of the teacher model, thereby learning the decision boundaries of the teacher model. The correspondences between the output sizes of each convolutional layer in the teacher and student models are shown in Table 5.

### 3.1. FPCN Model Effect

As shown in Table 6, we compared the method proposed in this article with basic image classification models and fine-grained classification models. Using the same rock image dataset, the proposed method achieved the best accuracy, reaching 93.1%. Compared with the next best models, PCA-Net and API-Net, both of which are based on contrastive learning, our method adds an unsupervised main body localization module, which achieves the filtering of invalid contrastive information, resulting in better effects. In comparison with MMAL-Net’s simultaneous localization of the main body and multi-part multi-view learning method, our approach considers the proportion of the object in the image and applies a soft mask based on the size of the ratio, supplementing the feature information when the target object’s localization is imprecise or the target object is small.

**Figure 6:**
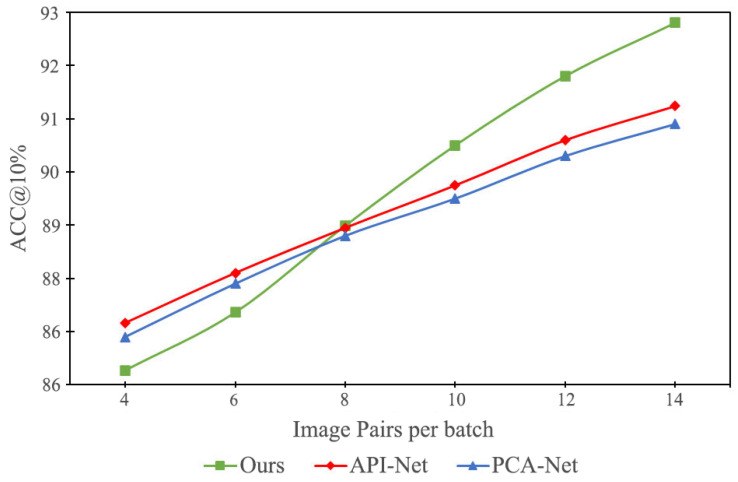
Effect of the number of sample pairs in a single batch for different models.

The impact of the number of image pairs in a single batch on the results can be seen in Figure 6; increasing the number of image pairs significantly improves the accuracy of all three models, indicating that the models learn more contrastive feature information and can more accurately categorize images that are difficult to distinguish. In the process of increasing contrast samples, the method proposed in this paper surpasses the other two contrast learning-based classification models. The possible reasons are as follows. First, when there are fewer contrast samples, the model receives limited feature information and the feature maps processed by the mask contain insufficient information, limiting the number of features extracted by the network. Second, when there are enough contrast samples, the model extracts a large number of contrast features, including some incorrect contrast features (such as the same background in the same class of images). Our proposed model can filter out more critical information from a large number of contrast features through object localization, thereby achieving better results.

### 3.2. Lightweight Distillation Effect

The results when training smaller student models using the model proposed in this paper as the teacher model are shown in Table 7. Each column in the figure represents various parameters of different models, including the total number of model parameters; the number of floating-point operations (FLOPs), which presents the floating point operations per second, shows the device’s requirements in terms of computing capacity. The inference time for a single sample presents the time between inputting the picture and outputting the classification result. The test set accuracy on the rock image dataset uses the knowledge distillation method by Hinton et al. and the knowledge distillation approach focusing on decision boundaries. The accuracy results of the small models distilled from the FPCN model are higher than those of the baseline models, achieving a balance between model efficiency and effectiveness to some extent.

The ResNet series (ResNet-18, ResNet-34, ResNet-50) and the MobileNet and ShuffleNet versions all achieve improved accuracy through distillation, which proves that the knowledge distillation technique can effectively transfer knowledge from the teacher model to the smaller student model while improving the latter’s performance. For the ResNet series, the improvement in accuracy due to distillation ranges from 0.6% to 1.4%. This shows that knowledge distillation is effective in improving performance even for relatively large models. In smaller models (e.g., MobileNet V1 and V2, ShuffleNetV2 1.5 and 0.5) the accuracy improvement is even more significant, ranging from 0.7% to 1.9%. This emphasizes the potential of knowledge distillation in compressing models and reducing computational resource requirements, especially for applications on mobile or edge devices. The inference time data shows that smaller models are more accurate and also run faster, which is important for applications that require fast response times. Among the ResNet models, the accuracy of ResNet18, ResNet34, and ResNet50 is 72.7%, 76.1%, and 77.1%, respectively, 0.9%, 1.4%, and 0.6% higher than the models without knowledge distillation (i.e., the models trained only with $L_{hard}$) and 0.6% higher than the proposed models; the largest ResNet50 model has reductions of nearly 50% in the number of parameters, number of floating-point operations, and inference time compared to the proposed FPCN model, which are 25.76 M, 16,529 M, and 7.5 s, respectively. Among the various models we tested, MobileNetV2 has the highest accuracy improvement of 1.9%, and the distillation models of MobileNetV1 and MobileNetV2 have the best overall performance, which is only one-tenth of the FPCN in terms of various resource consumption indexes. Moreover, the accuracy is 5% higher than that of the two ShuffleNet networks. For ShuffleNetV2 1.5 and ShuffleNetV2 0.5, although the resource consumption is very small and the number of parameters is only 2.4 M and 0.3 M, the model accuracy is lower and the distillation effect is not significant, which may be due to the fact that the model with the number of parameters may not be able to fit more complex decision boundaries, meaning that the proposed optimization of activation boundaries leads to greater variation of the decision boundaries and leading to part of the originally correct samples being misclassified.

To further demonstrate the effectiveness of closer decision boundaries, the relationship between the similarity of neuron activation in the last layer of each convolutional layer of ResNet and the final classification accuracy is listed in Table 8. It can be seen from the table that there is a positive overall correlation between neuron activation similarity and accuracy. For the ResNet-18 and ResNet-34 models, the neuron activation similarities for the shallowest and deepest layers reach 95.7%, 98.6%, 72.7%, and 76.1%, respectively, each showing nearly 1% improvement compared to the knowledge distillation methods without optimized activation boundaries.

In all models, the method of knowledge distillation with decision boundary (KD+DB) significantly improved the neuron similarity across all convolutional layers ($conv_{2}$, $conv_{3}$, $conv_{4}$, $conv_{5}$) compared to using knowledge distillation alone. This indicates that the KD+DB method can more effectively transfer the feature representation ability of the teacher model to the student model, more closely mimicking the teacher model’s behavior at deeper levels.

In terms of accuracy, all models using the KD+DB method outperformed those using only the KD method. This shows that by better simulating the neuronal behavior of the teacher model, the student model can learn the decision boundaries of the teacher model more accurately, resulting in improved overall performance of the model.

In comparing the models with different structures, as the model depth of the ResNet series increases (from ResNet-18 to ResNet-34), both the neuron similarity and accuracy improve using either the KD or KD+DB method. This suggests that deeper models are better able to learn and simulate the characteristics of the teacher model during knowledge distillation. For MobileNetV2 and ShuffleNetV2, the improvement in neuron similarity is particularly significant compared to the ResNet series when using the KD+DB method, which is especially the case in deeper convolutional layers. This indicates that the KD+DB method may enhance model performance more effectively for lightweight models with higher computational efficiency.

**Figure 7:**
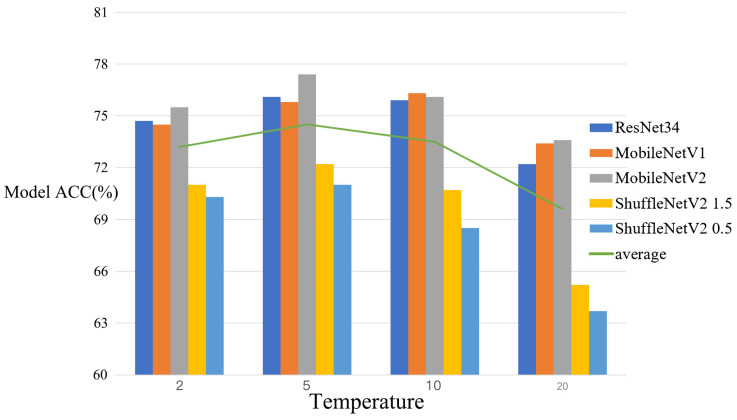
Effect of different distillation temperatures on the accuracy of different models.

The temperature used during knowledge distillation for fine-grained tasks also impacts the outcomes. The temperature level alters the degree of attention the student model pays to negative labels during the training process; at lower temperatures, less attention is paid to negative labels, especially those significantly below the average, while at higher temperatures the values associated with negative labels are relatively higher, leading the student model to pay more attention to negative labels. Indeed, negative labels can contain certain types of information, particularly those with values significantly above the average; however, due to the training process used by the FPCN, the negative label part contains more noise, with lower values of the negative label indicating less reliable information. Therefore, the choice of temperature has a significant impact on the distillation outcomes. For fine-grained classification tasks, the impact of negative labels is mainly divided into two categories. In the first category, the negative label scores assigned by the FPCN model are very close to the positive label scores. This indicates ne of two scenarios; first, the existence of similar samples between different classes, providing crucial detail information for fine-grained classification decisions, in which case paying more attention to negative label information aids training; or second, misclassification by the FPCN teacher model, resulting in high negative label scores, in which case overemphasis leads to incorrect training information. In the second category, the negative label scores assigned by the FPCN model have a large gap compared to the positive label scores, in which case the impact of negative labels is smaller. For scenarios in the first category, choosing different temperatures can enhance the effectiveness of knowledge distillation in fine-grained sample contexts. Figure 7 shows the impact of knowledge distillation at different temperatures on model performance.

## 4. Conclusions

In this paper, a fine-grained image classification model based on deep learning is proposed to solve the problem of low classification accuracy on the part of existing methods for fine-grained rock image classification by mobile lightweight models. The proposed model is used to develop an improved lightweight model for mobile applications; specifically, a Feature-map Positioning and Contrastive Learning Network (FPCN) is proposed for weakly supervised fine-grained rock image recognition, guiding the generation of lightweight classification models on mobile devices by shaping the transfer decision boundaries. Our findings indicate that focusing on knowledge distillation around the decision boundaries enhances the accuracy of lightweight models.

## Figures and Tables

**Table 1:** Rock sample classification approaches.

Rock Classification Algorithm	Advantages	Disadvantages	
SVM Classification	strong generalization ability	cannot deal with mass of rock image samples	
Location-Classification CNN	excludes background noise	additional annotation	larger time and space costs
Contrast Learning-based Classification	captures detailed features	irrelevant comparison features lead to classification mistakes	
FPCN (ours)	combines advantages of the above two models		
Distilled Lightweight Model by FPCN (ours)	balances accuracy and consumption		

**Table 2:** Structure of the transformation layers.

Connect Layers	Input	Output	Kernal
Connector1	$H_{1}\times W_{1}\times C_{1s}$	$H_{1}\times W_{1}\times C_{1t}$	1×1,stride=1,padding=0
Connector2	$H_{1}\times W_{1}\times C_{2s}$	$H_{1}\times W_{1}\times C_{2t}$	
Connector3	$H_{1}\times W_{1}\times C_{3s}$	$H_{1}\times W_{1}\times C_{3t}$	
Connector4	$H_{1}\times W_{1}\times C_{4s}$	$H_{1}\times W_{1}\times C_{4t}$	

**Table 3:** FGVC-ROCK dataset.

Rock Type	Number
Dolomite	7924
Marble	11,658
Basalt	19,678
All	39,620

**Table 4:** Transforms used in training and testing.

Train			Test		
Operation	Input Size	Output Size	Operation	Input Size	Output Size
resize	Original Image Size	(512,512,3)	resize	Original Image Size	(512,512,3)
Random Cropping	(512,512,3)	(448,448,3)	Center Cropping	(512,512,3)	(448,448,3)
Random horizontal Flipping	(448,448,3)	(448,448,3)			
Normalization	(448,448,3)	(448,448,3)	Normalization	(448,448,3)	(448,448,3)

**Table 5:** Connection of convolutional layer.

Model	Block1	Block2	Block3	Block4
ResNet-101 (teacher)	conv_2	conv_3	conv_4	conv_5
ResNet-18 ResNet-34	conv_2	conv_3	conv_4	conv_5
MobileNetV1 MobileNetV2	Conv dw/s2(1)	Conv dw/s2(2)	Conv dw/s2(3)	Conv dw/s2(4)
ShuffleNetV1 ShuffleNetV2	Conv1 MaxPool	Stage2	Stage3	Stage4
Output Size	112×112	56×56	28×28	14×14

**Table 6:** Comparison of different fine-grained classification models.

Models	Training Accuracy	Test Accuracy	Backbone
Bilinear CNN (2015)	85.4	85.3	VGG16
CIN (2020)	93.2	89.8	ResNet-101
PCA-Net (2021)	94.9	90.8	ResNet-101
API-Net (2020)	99.8	91.2	ResNet-101
MMAL-Net (2021)	99.7	91.9	ResNet-50
FPCN	99.8	93.1	ResNet-101

**Table 7:** Distillation improvements on lightweight models.

Model	(Teacher) FPCN	ResNet-18	ResNet-34	ResNet-50	MobileNet V1	MobileNet V2	ShuffleNet V2 1.5	ShuffleNet V2 0.5
Params (Million)	46.06 M	11.89 M	22.00 M	25.76 M	4.2 M	3.4 M	2.4 M	0.3 M
FLOPS	28,210 M	7294 M	14,713 M	16,529 M	569 M	326 M	303 M	43 M
Inference Time (s)	13.98	2.61	5.63	7.50	1.13	0.78	0.68	0.16
Baseline Accuracy (%)	93.1	71.8	74.7	76.5	74.5	75.5	71.4	70.3
Distilled Accuracy (%)		72.7 (+0.9)	76.1 (+1.4)	77.1 (+0.6)	76.3 (+1.8)	77.4 (+1.9)	72.2 (+0.8)	71.0 (+0.7)

**Table 8:** Neuron similarity in lightweight models.

Models	Similarity(%)				Accuracy (%)
	conv_2	conv_3	conv_4	conv_5	
KD ResNet-18	93.3	82.6	70.3	59.8	71.8
KD+DB ResNet-18	95.7	93.9	89.0	76.5	72.7
KD ResNet-34	96.7	96.5	93.9	83.3	74.7
KD+DB ResNet-34	98.6	98.0	95.1	85.6	76.1
KD MobileNetV2	98.1	86.9	73.9	73.4	75.5
KD+DB MobileNetV2	99.1	98.9	95.7	82.2	77.4
KD ShuffleNetV2 1.5	92.4	81.8	69.6	59.2	71.4
KD+DB ShuffleNetV2 1.5	94.7	93.0	88.1	75.7	72.2

## Data Availability

The data presented in this study are openly available under Feature Positioning Contrast Network at https://doi.org/10.5281/zenodo.10099204 (accessed on 15 June 2024).
